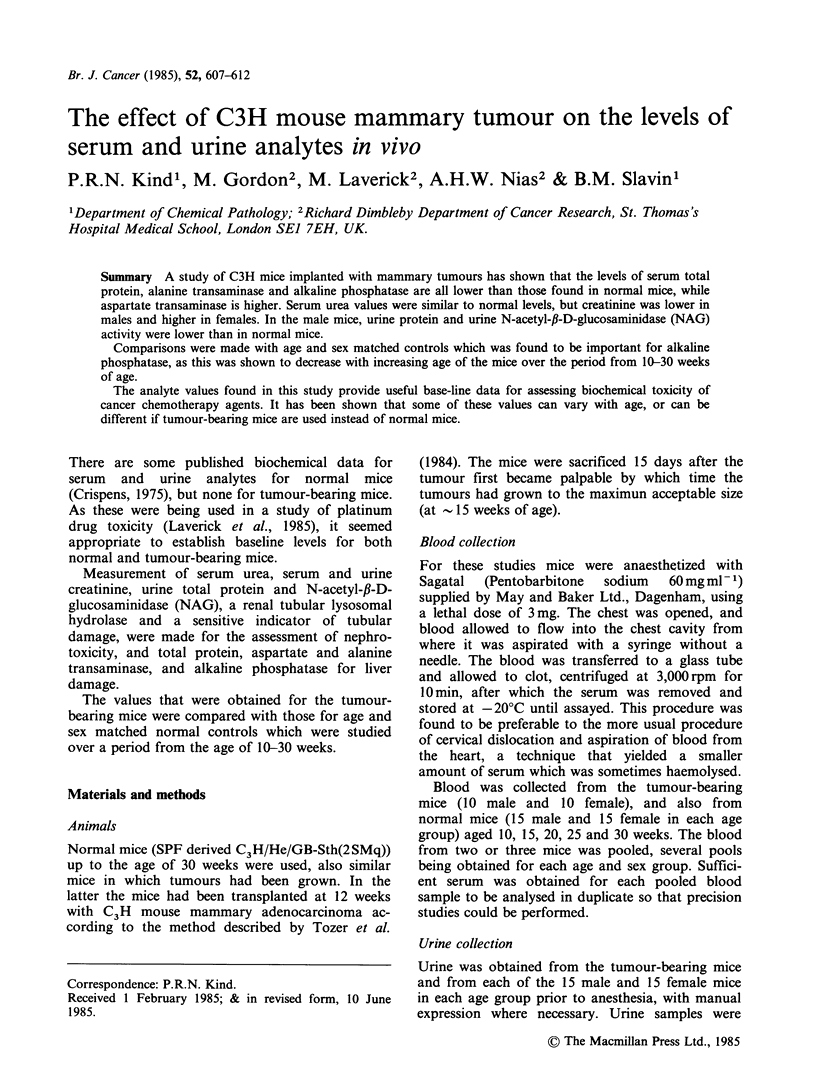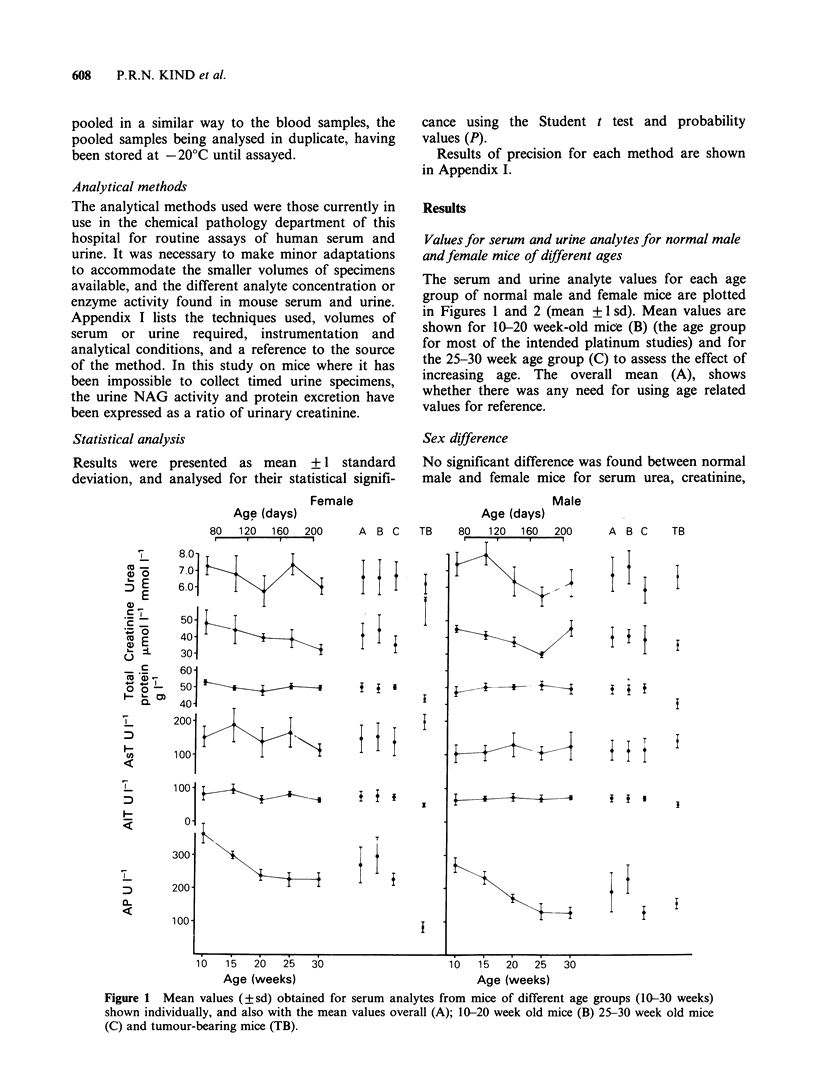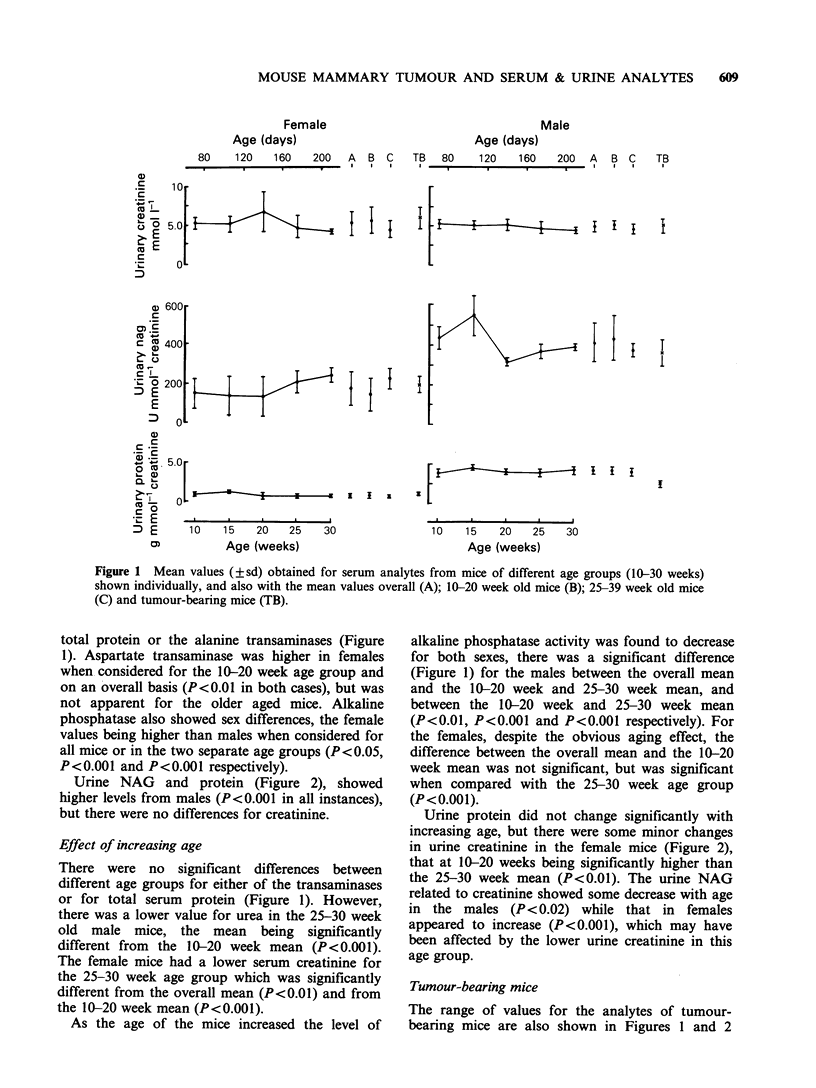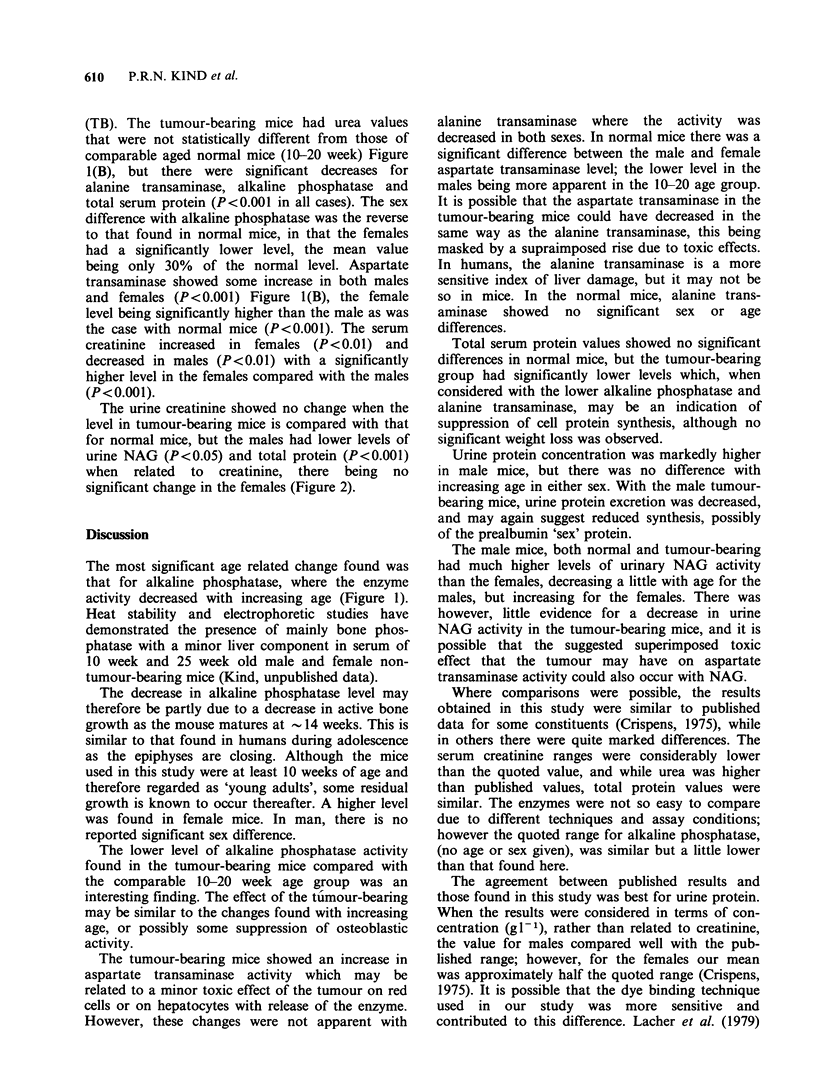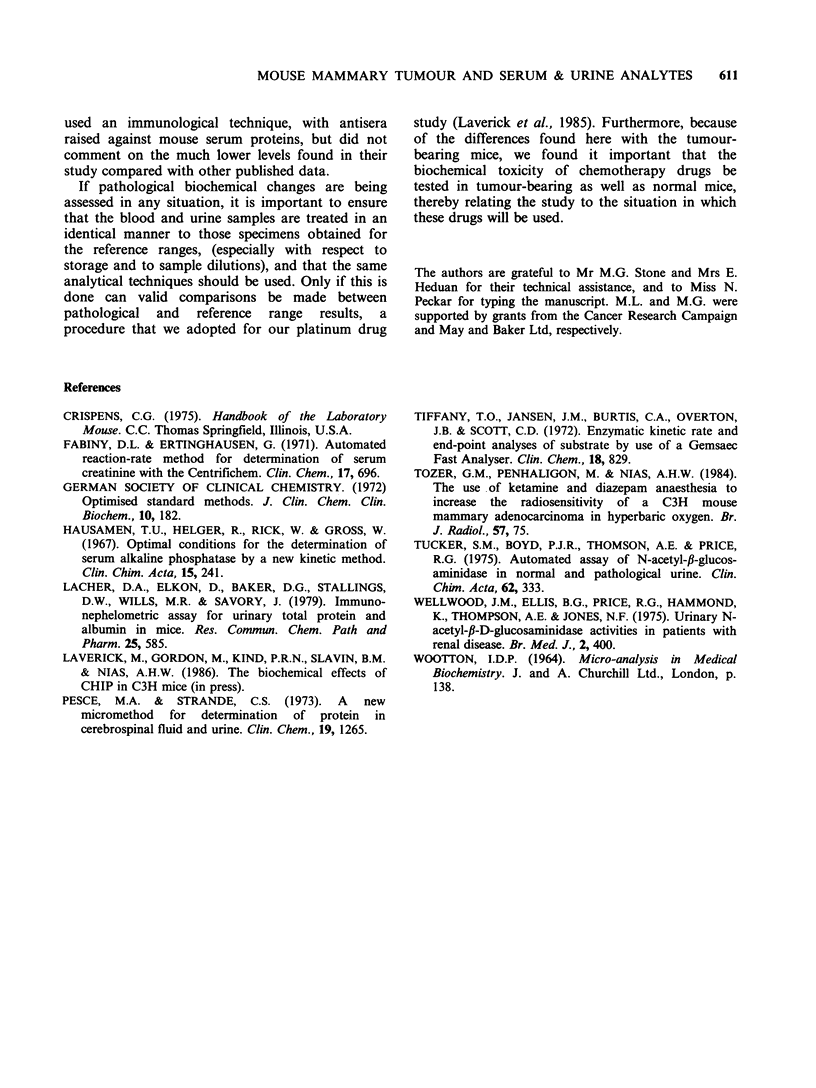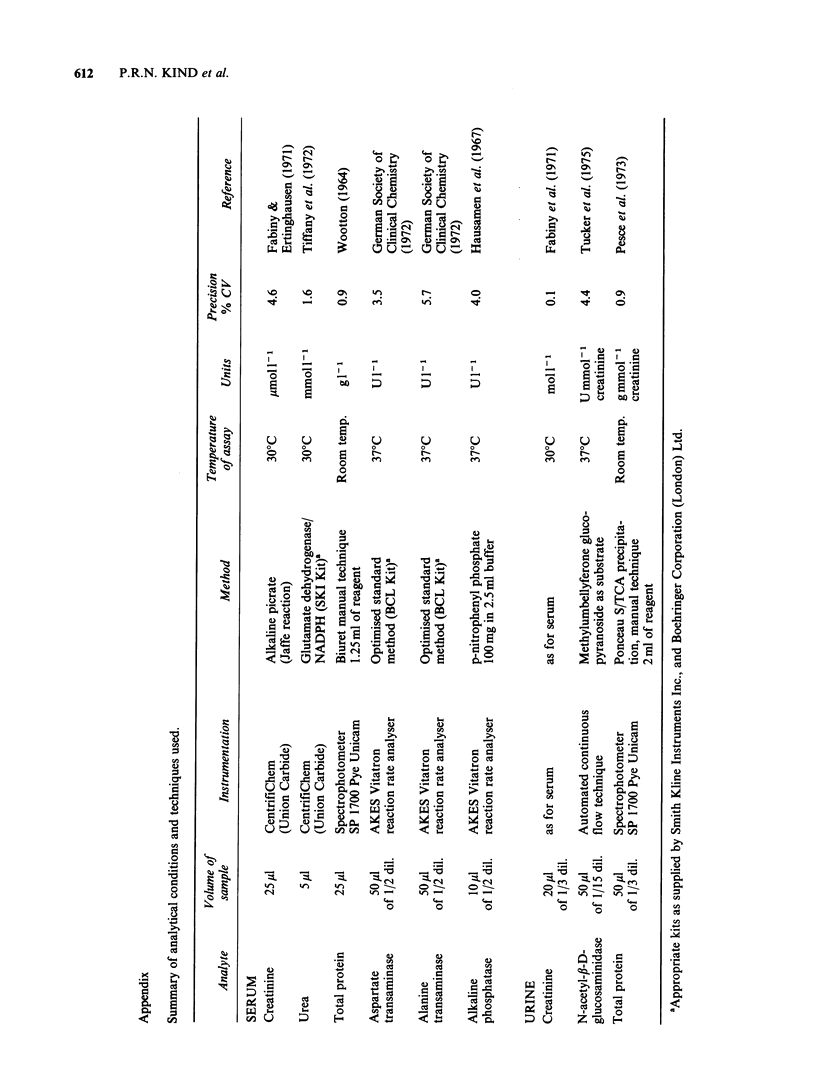# The effect of C3H mouse mammary tumour on the levels of serum and urine analytes in vivo.

**DOI:** 10.1038/bjc.1985.233

**Published:** 1985-10

**Authors:** P. R. Kind, M. Gordon, M. Laverick, A. H. Nias, B. M. Slavin

## Abstract

A study of C3H mice implanted with mammary tumours has shown that the levels of serum total protein, alanine transaminase and alkaline phosphatase are all lower than those found in normal mice, while aspartate transaminase is higher. Serum urea values were similar to normal levels, but creatinine was lower in males and higher in females. In the male mice, urine protein and urine N-acetyl-beta-D-glucosaminidase (NAG) activity were lower than in normal mice. Comparisons were made with age and sex matched controls which was found to be important for alkaline phosphatase, as this was shown to decrease with increasing age of the mice over the period from 10-30 weeks of age. The analyte values found in this study provide useful base-line data for assessing biochemical toxicity of cancer chemotherapy agents. It has been shown that some of these values can vary with age, or can be different if tumour-bearing mice are used instead of normal mice.


*Br. J. Cancer* (1985), **52**, 607–612

### The effect of C3H mouse mammary tumour on the levels of serum and urine analytes *in vivo*

P.R.N. Kind[1], M. Gordon[2], M. Laverick[2], A.H.W. Nias[2] & B.M. Slavin[1]

[1]*Department of Chemical Pathology;* [2]*Richard Dimbleby Department of Cancer Research, St. Thomas's Hospital Medical School, London SE1 7EH, UK.*

**Summary** A study of C3H mice implanted with mammary tumours has shown that the levels of serum total protein, alanine transaminase and alkaline phosphatase are all lower than those found in normal mice, while aspartate transaminase is higher. Serum urea values were similar to normal levels, but creatinine was lower in males and higher in females. In the male mice, urine protein and urine N-acetyl-$\beta$-D-glucosaminidase (NAG) activity were lower than in normal mice.

Comparisons were made with age and sex matched controls which was found to be important for alkaline phosphatase, as this was shown to decrease with increasing age of the mice over the period from 10–30 weeks of age.

The analyte values found in this study provide useful base-line data for assessing biochemical toxicity of cancer chemotherapy agents. It has been shown that some of these values can vary with age, or can be different if tumour-bearing mice are used instead of normal mice.

There are some published biochemical data for serum and urine analytes for normal mice (Crispens, 1975), but none for tumour-bearing mice. As these were being used in a study of platinum drug toxicity (Laverick *et al.*, 1985), it seemed appropriate to establish baseline levels for both normal and tumour-bearing mice.

Measurement of serum urea, serum and urine creatinine, urine total protein and N-acetyl-$\beta$-D-glucosaminidase (NAG), a renal tubular lysosomal hydrolase and a sensitive indicator of tubular damage, were made for the assessment of nephrotoxicity, and total protein, aspartate and alanine transaminase, and alkaline phosphatase for liver damage.

The values that were obtained for the tumour-bearing mice were compared with those for age and sex matched normal controls which were studied over a period from the age of 10–30 weeks.

### Materials and methods

### *Animals*

Normal mice (SPF derived $C_3H$/He/GB-Sth(2SMq)) up to the age of 30 weeks were used, also similar mice in which tumours had been grown. In the latter the mice had been transplanted at 12 weeks with $C_3H$ mouse mammary adenocarcinoma according to the method described by Tozer *et al.* (1984). The mice were sacrificed 15 days after the tumour first became palpable by which time the tumours had grown to the maximun acceptable size (at $\sim$15 weeks of age).

### *Blood collection*

For these studies mice were anaesthetized with Sagatal (Pentobarbitone sodium 60 mg ml$^{-1}$) supplied by May and Baker Ltd., Dagenham, using a lethal dose of 3 mg. The chest was opened, and blood allowed to flow into the chest cavity from where it was aspirated with a syringe without a needle. The blood was transferred to a glass tube and allowed to clot, centrifuged at 3,000 rpm for 10 min, after which the serum was removed and stored at −20°C until assayed. This procedure was found to be preferable to the more usual procedure of cervical dislocation and aspiration of blood from the heart, a technique that yielded a smaller amount of serum which was sometimes haemolysed.

Blood was collected from the tumour-bearing mice (10 male and 10 female), and also from normal mice (15 male and 15 female in each age group) aged 10, 15, 20, 25 and 30 weeks. The blood from two or three mice was pooled, several pools being obtained for each age and sex group. Sufficient serum was obtained for each pooled blood sample to be analysed in duplicate so that precision studies could be performed.

### *Urine collection*

Urine was obtained from the tumour-bearing mice and from each of the 15 male and 15 female mice in each age group prior to anesthesia, with manual expression where necessary. Urine samples were

Correspondence: P.R.N. Kind.
Received 1 February 1985; & in revised form, 10 June 1985.

© The Macmillan Press Ltd., 1985

pooled in a similar way to the blood samples, the pooled samples being analysed in duplicate, having been stored at −20°C until assayed.

### *Analytical methods*

The analytical methods used were those currently in use in the chemical pathology department of this hospital for routine assays of human serum and urine. It was necessary to make minor adaptations to accommodate the smaller volumes of specimens available, and the different analyte concentration or enzyme activity found in mouse serum and urine. Appendix I lists the techniques used, volumes of serum or urine required, instrumentation and analytical conditions, and a reference to the source of the method. In this study on mice where it has been impossible to collect timed urine specimens, the urine NAG activity and protein excretion have been expressed as a ratio of urinary creatinine.

### *Statistical analysis*

Results were presented as mean ±1 standard deviation, and analysed for their statistical significance using the Student *t* test and probability values (*P*).

Results of precision for each method are shown in Appendix I.

### Results

### *Values for serum and urine analytes for normal male and female mice of different ages*

The serum and urine analyte values for each age group of normal male and female mice are plotted in Figures 1 and 2 (mean ±1 sd). Mean values are shown for 10–20 week-old mice (B) (the age group for most of the intended platinum studies) and for the 25–30 week age group (C) to assess the effect of increasing age. The overall mean (A), shows whether there was any need for using age related values for reference.

### *Sex difference*

No significant difference was found between normal male and female mice for serum urea, creatinine,

**Figure 1** Mean values (±sd) obtained for serum analytes from mice of different age groups (10–30 weeks) shown individually, and also with the mean values overall (A); 10–20 week old mice (B) 25–30 week old mice (C) and tumour-bearing mice (TB).

**Figure 1** Mean values (±sd) obtained for serum analytes from mice of different age groups (10–30 weeks) shown individually, and also with the mean values overall (A); 10–20 week old mice (B); 25–39 week old mice (C) and tumour-bearing mice (TB).

total protein or the alanine transaminases (Figure 1). Aspartate transaminase was higher in females when considered for the 10–20 week age group and on an overall basis ($P<0.01$ in both cases), but was not apparent for the older aged mice. Alkaline phosphatase also showed sex differences, the female values being higher than males when considered for all mice or in the two separate age groups ($P<0.05$, $P<0.001$ and $P<0.001$ respectively).

Urine NAG and protein (Figure 2), showed higher levels from males ($P<0.001$ in all instances), but there were no differences for creatinine.

*Effect of increasing age*

There were no significant differences between different age groups for either of the transaminases or for total serum protein (Figure 1). However, there was a lower value for urea in the 25–30 week old male mice, the mean being significantly different from the 10–20 week mean ($P<0.001$). The female mice had a lower serum creatinine for the 25–30 week age group which was significantly different from the overall mean ($P<0.01$) and from the 10–20 week mean ($P<0.001$).

As the age of the mice increased the level of alkaline phosphatase activity was found to decrease for both sexes, there was a significant difference (Figure 1) for the males between the overall mean and the 10–20 week and 25–30 week mean, and between the 10–20 week and 25–30 week mean ($P<0.01$, $P<0.001$ and $P<0.001$ respectively). For the females, despite the obvious aging effect, the difference between the overall mean and the 10–20 week mean was not significant, but was significant when compared with the 25–30 week age group ($P<0.001$).

Urine protein did not change significantly with increasing age, but there were some minor changes in urine creatinine in the female mice (Figure 2), that at 10–20 weeks being significantly higher than the 25–30 week mean ($P<0.01$). The urine NAG related to creatinine showed some decrease with age in the males ($P<0.02$) while that in females appeared to increase ($P<0.001$), which may have been affected by the lower urine creatinine in this age group.

*Tumour-bearing mice*

The range of values for the analytes of tumour-bearing mice are also shown in Figures 1 and 2

(TB). The tumour-bearing mice had urea values that were not statistically different from those of comparable aged normal mice (10–20 week) Figure 1(B), but there were significant decreases for alanine transaminase, alkaline phosphatase and total serum protein ($P<0.001$ in all cases). The sex difference with alkaline phosphatase was the reverse to that found in normal mice, in that the females had a significantly lower level, the mean value being only 30% of the normal level. Aspartate transaminase showed some increase in both males and females ($P<0.001$) Figure 1(B), the female level being significantly higher than the male as was the case with normal mice ($P<0.001$). The serum creatinine increased in females ($P<0.01$) and decreased in males ($P<0.01$) with a significantly higher level in the females compared with the males ($P<0.001$).

The urine creatinine showed no change when the level in tumour-bearing mice is compared with that for normal mice, but the males had lower levels of urine NAG ($P<0.05$) and total protein ($P<0.001$) when related to creatinine, there being no significant change in the females (Figure 2).

### Discussion

The most significant age related change found was that for alkaline phosphatase, where the enzyme activity decreased with increasing age (Figure 1). Heat stability and electrophoretic studies have demonstrated the presence of mainly bone phosphatase with a minor liver component in serum of 10 week and 25 week old male and female non-tumour-bearing mice (Kind, unpublished data).

The decrease in alkaline phosphatase level may therefore be partly due to a decrease in active bone growth as the mouse matures at ~14 weeks. This is similar to that found in humans during adolescence as the epiphyses are closing. Although the mice used in this study were at least 10 weeks of age and therefore regarded as 'young adults', some residual growth is known to occur thereafter. A higher level was found in female mice. In man, there is no reported significant sex difference.

The lower level of alkaline phosphatase activity found in the tumour-bearing mice compared with the comparable 10–20 week age group was an interesting finding. The effect of the tumour-bearing may be similar to the changes found with increasing age, or possibly some suppression of osteoblastic activity.

The tumour-bearing mice showed an increase in aspartate transaminase activity which may be related to a minor toxic effect of the tumour on red cells or on hepatocytes with release of the enzyme. However, these changes were not apparent with alanine transaminase where the activity was decreased in both sexes. In normal mice there was a significant difference between the male and female aspartate transaminase level; the lower level in the males being more apparent in the 10–20 age group. It is possible that the aspartate transaminase in the tumour-bearing mice could have decreased in the same way as the alanine transaminase, this being masked by a supraimposed rise due to toxic effects. In humans, the alanine transaminase is a more sensitive index of liver damage, but it may not be so in mice. In the normal mice, alanine transaminase showed no significant sex or age differences.

Total serum protein values showed no significant differences in normal mice, but the tumour-bearing group had significantly lower levels which, when considered with the lower alkaline phosphatase and alanine transaminase, may be an indication of suppression of cell protein synthesis, although no significant weight loss was observed.

Urine protein concentration was markedly higher in male mice, but there was no difference with increasing age in either sex. With the male tumour-bearing mice, urine protein excretion was decreased, and may again suggest reduced synthesis, possibly of the prealbumin 'sex' protein.

The male mice, both normal and tumour-bearing had much higher levels of urinary NAG activity than the females, decreasing a little with age for the males, but increasing for the females. There was however, little evidence for a decrease in urine NAG activity in the tumour-bearing mice, and it is possible that the suggested superimposed toxic effect that the tumour may have on aspartate transaminase activity could also occur with NAG.

Where comparisons were possible, the results obtained in this study were similar to published data for some constituents (Crispens, 1975), while in others there were quite marked differences. The serum creatinine ranges were considerably lower than the quoted value, and while urea was higher than published values, total protein values were similar. The enzymes were not so easy to compare due to different techniques and assay conditions; however the quoted range for alkaline phosphatase, (no age or sex given), was similar but a little lower than that found here.

The agreement between published results and those found in this study was best for urine protein. When the results were considered in terms of concentration ($g\,l^{-1}$), rather than related to creatinine, the value for males compared well with the published range; however, for the females our mean was approximately half the quoted range (Crispens, 1975). It is possible that the dye binding technique used in our study was more sensitive and contributed to this difference. Lacher *et al.* (1979)

used an immunological technique, with antisera raised against mouse serum proteins, but did not comment on the much lower levels found in their study compared with other published data.

If pathological biochemical changes are being assessed in any situation, it is important to ensure that the blood and urine samples are treated in an identical manner to those specimens obtained for the reference ranges, (especially with respect to storage and to sample dilutions), and that the same analytical techniques should be used. Only if this is done can valid comparisons be made between pathological and reference range results, a procedure that we adopted for our platinum drug study (Laverick *et al.*, 1985). Furthermore, because of the differences found here with the tumour-bearing mice, we found it important that the biochemical toxicity of chemotherapy drugs be tested in tumour-bearing as well as normal mice, thereby relating the study to the situation in which these drugs will be used.

The authors are grateful to Mr M.G. Stone and Mrs E. Heduan for their technical assistance, and to Miss N. Peckar for typing the manuscript. M.L. and M.G. were supported by grants from the Cancer Research Campaign and May and Baker Ltd, respectively.

**References**

CRISPENS, C.G. (1975). *Handbook of the Laboratory Mouse*. C.C. Thomas Springfield, Illinois, U.S.A.

FABINY, D.L. & ERTINGHAUSEN, G. (1971). Automated reaction-rate method for determination of serum creatinine with the Centrifichem. *Clin. Chem.*, **17**, 696.

GERMAN SOCIETY OF CLINICAL CHEMISTRY. (1972) Optimised standard methods. *J. Clin. Chem. Clin. Biochem.*, **10**, 182.

HAUSAMEN, T.U., HELGER, R., RICK, W. & GROSS, W. (1967). Optimal conditions for the determination of serum alkaline phosphatase by a new kinetic method. *Clin. Chim. Acta*, **15**, 241.

LACHER, D.A., ELKON, D., BAKER, D.G., STALLINGS, D.W., WILLS, M.R. & SAVORY, J. (1979). Immunonephelometric assay for urinary total protein and albumin in mice. *Res. Commun. Chem. Path and Pharm.* **25**, 585.

LAVERICK, M., GORDON, M., KIND, P.R.N., SLAVIN, B.M. & NIAS, A.H.W. (1986). The biochemical effects of CHIP in C3H mice (in press).

PESCE, M.A. & STRANDE, C.S. (1973). A new micromethod for determination of protein in cerebrospinal fluid and urine. *Clin. Chem.*, **19**, 1265.

TIFFANY, T.O., JANSEN, J.M., BURTIS, C.A., OVERTON, J.B. & SCOTT, C.D. (1972). Enzymatic kinetic rate and end-point analyses of substrate by use of a Gemsaec Fast Analyser. *Clin. Chem.*, **18**, 829.

TOZER, G.M., PENHALIGON, M. & NIAS, A.H.W. (1984). The use of ketamine and diazepam anaesthesia to increase the radiosensitivity of a C3H mouse mammary adenocarcinoma in hyperbaric oxygen. *Br. J. Radiol.*, **57**, 75.

TUCKER, S.M., BOYD, P.J.R., THOMSON, A.E. & PRICE, R.G. (1975). Automated assay of N-acetyl-β-glucosaminidase in normal and pathological urine. *Clin. Chim. Acta*, **62**, 333.

WELLWOOD, J.M., ELLIS, B.G., PRICE, R.G., HAMMOND, K., THOMPSON, A.E. & JONES, N.F. (1975). Urinary N-acetyl-β-D-glucosaminidase activities in patients with renal disease. *Br. Med. J.*, **2**, 400.

WOOTTON, I.D.P. (1964). *Micro-analysis in Medical Biochemistry*. J. and A. Churchill Ltd., London, p. 138.